# Accelerator or Brake: Immune Regulators in Malaria

**DOI:** 10.3389/fcimb.2020.610121

**Published:** 2020-12-10

**Authors:** Chunmei Cai, Zhiqiang Hu, Xiao Yu

**Affiliations:** ^1^ Research Center for High Altitude Medicine, School of Medical, Qinghai University, Xining, China; ^2^ Key Laboratory of Application and Foundation for High Altitude Medicine Research in Qinghai Province, Qinghai University, Xining, China; ^3^ Department of Immunology, School of Basic Medical Sciences, Southern Medical University, Guangzhou, China; ^4^ Guangdong Provincial Key Lab of Single Cell Technology and Application, Southern Medical University, Guangzhou, China

**Keywords:** malaria, immune regulators, immune responses, type I interferon, signaling mechanisms, protective immunity

## Abstract

Malaria is a life-threatening infectious disease, affecting over 250 million individuals worldwide each year, eradicating malaria has been one of the greatest challenges to public health for a century. Growing resistance to anti-parasitic therapies and lack of effective vaccines are major contributing factors in controlling this disease. However, the incomplete understanding of parasite interactions with host anti-malaria immunity hinders vaccine development efforts to date. Recent studies have been unveiling the complexity of immune responses and regulators against *Plasmodium* infection. Here, we summarize our current understanding of host immune responses against *Plasmodium*-derived components infection and mainly focus on the various regulatory mechanisms mediated by recent identified immune regulators orchestrating anti-malaria immunity.

## Introduction

Malaria, caused by *Plasmodium*, is one of the deadly infectious diseases worldwide (Battle et al., 2015; Howes et al., 2016). According to the World Health Organization report (WHO, 2019), this infectious disease affected up to 260 million individuals, and caused about half a million deaths in 2018. When female *Anopheles* mosquitoes inject *Plasmodium* sporozoites into mammalian hosts skin, the malaria infection is initiated, leading to a complex life cycle (Ross, 1896; Grassi et al., 1899). After that, the sporozoites travel through the bloodstream to the liver (Tavares et al., 2013). Once sporozoites reach the liver, they infect hepatocytes and replicate to about 30,000 merozoites, which are then released back into the peripheral blood (Mota et al., 2001). Merozoites infect red blood cells (RBCs) rapidly, and the repeated cycle, including invasions, replication and release, leads to exponential growth of parasites and disease (Amino et al., 2006; Sturm et al., 2006). The complex and multi-staged life cycle of malaria parasites evokes a slow development of immunity to protect parasites from being eliminated.

Over the past decade, the malaria disease, death, and transmission rates significantly decreased in most endemic countries. However, this stunning progress has been halted by emergence of drug resistance (WHO, 2019). Besides, the lack of an effective vaccine has been a major constraint in the prevention of malaria infection, which largely due to the underlying mechanism of host-parasite interactions is poorly understood (Riley and Stewart, 2013; Arama and Troye-Blomberg, 2014; Ouattara and Laurens, 2015). Malaria infection triggers a systemic immune response, and results in the increase of inflammatory cytokines production that leads to parasite elimination or disease (Stevenson and Riley, 2004; Parroche et al., 2007; Coban et al., 2010; Sharma et al., 2011; Gazzinelli et al., 2014; Kalantari et al., 2014; Wu et al., 2014; Mendonca and Barral-Netto, 2015). A fine-tuned regulation of immune responses is crucial for developing protective immunity to effectively eliminate malaria parasites and preventing overreacted damage to host. Hence, a comprehensive understanding of the molecular and regulatory mechanisms that modulate the immunity against *Plasmodium* is pivotal to develop effective therapeutics and vaccines.

In this Review, we briefly summarize the activation and function of immune responses to malaria invasion, and mainly focus on the immune regulators in anti-malaria immunity. We describe parasites recognition by host, and the following initiation as well as function of host immune responses. Additionally, we discuss how the known regulators manipulate above immune activation and direct our attention on our group’s findings. These include that an early spike of type I interferon (IFN-I) is protective against blood stages in *Plasmodium* infection, which is modulated by CD40, SOCS1, FOSL1, MARCH1, as well as RTP4, regulators identified by our group and collaborators.

## Anti-Malaria Immunity

**Figure 1:**
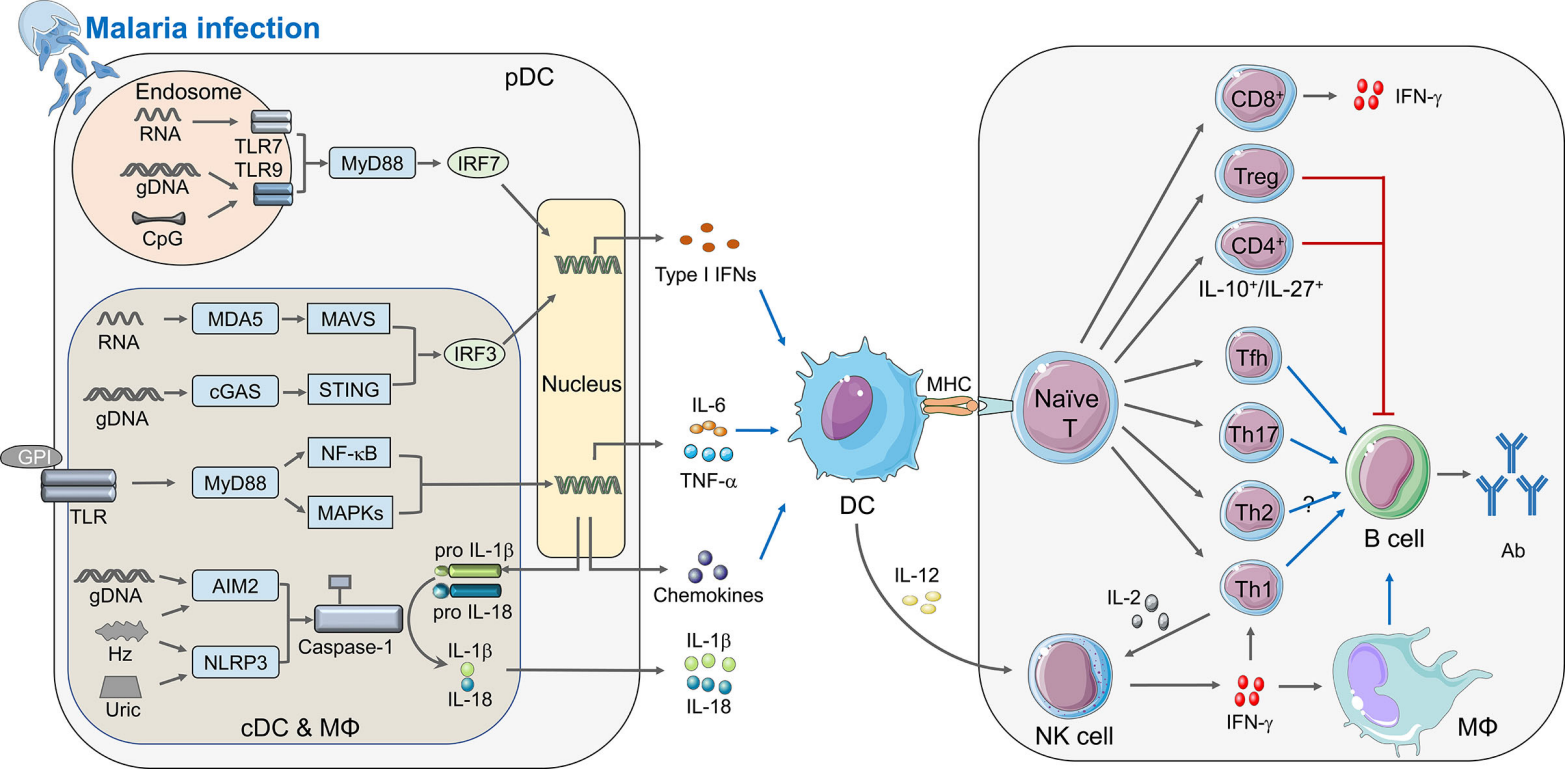
Immune responses elicited by *Plasmodium* infection. During malaria infection, different PAMPs secreted from merozoites can be sensed by PRRs and activate the innate immunity (left panel). cDCs, macrophages, and pDCs are the crucial innate immune cells to defend malaria infection. Within the cytosol of these cells, pathogenic RNA interacts with MDA5 and recruits the adaptor protein MAVS, and *Plasmodium* gDNA can be detected by cGAS or other DNA sensors to activate adaptor protein STING. Both MAVS and STING could recruit serine/threonine-protein kinase TBK1 to phosphorylate IRF3 which translocate to nucleus and induce the expression of IFN-I. Furthermore, parasitic nucleic acid gDNA can also be sensed by inflammasome sensors AIM2, whereas haemozoin and uric acid activates NLRP3, leading to activation of inflammasomes and Caspase-1, which cleave pro-IL-1β and pro-IL-18 to form mature IL-1β and IL-18. Besides, parasite glycosylphosphatidylinositol (GPI) anchors to TLRs, including TLR2-TLR6 or TLR1-TLR2 heterodimers and TLR4 homodimers. TLRs signal transduces through MyD88, which finally causes the activation of NF-κB and MAPKs, and induces the secretion of pro-inflammatory cytokines, such as TNF-α and IL-6, as well as chemokines. Specifically, both CpG and hemozoin-combined gDNA can induce TLR9 translocation, and TLR7 can sense parasite RNA in the endosome of pDCs during the early infection stage, TLR9 as well as TLR7 recruit adaptor protein MyD88 and kinases TRAFs to phosphorylate IRF7 and induce the early robust production of IFN-I. After the innate immune responses, DC acts as a vital APC receiving stimulation via cytokines described upon, is activated and presents the antigens to naïve T cell through combination of MHC and TCR, which builds a bridge between innate and adaptive immunity. During adaptive immunity (right panel), naïve T cells differentiate into different subtypes with unique functions in anti-malaria immunity. Tfh, Th1, and Th17 could facilitate function of B cells, while Treg and IL-10^+^/IL-27^+^ CD4^+^ T cell can suppress B cell function; CD8^+^ T cells mainly express IFN-γ, and leaving function of Th2 is still uncertain. Activated DCs can also secrete IL-12 to promote the expression of IFN-γ from NK cell, which can enhance function of Th1 and macrophage thus help B cells to secrete antibody. Besides, IL-2 produced by Th1 can help NK cell to kill parasites. The diagram depicts a simplified version of indicated signaling pathway and immune cells involved in anti-malaria immunity. The abbreviations are defined in footnote.

Malaria infection is initiated by the bite of mosquitoes carrying *Plasmodium* sporozoites. Those sporozoites target liver and infect hepatocytes when they enter the bloodstream at the first step, referred to as the liver stage. After that, merozoites released from the infected hepatocytes invade RBCs, which is called the blood stage infection. During infection, the host immune system senses the invading of *Plasmodium* at both liver stage and blood stage, and initiates the innate immune responses to produce cytokines and chemokines, which further activates antigen presenting cells to bridge the innate and adaptive immunity against malaria (**Figure 1**).

### Parasite Sensing

Host detects *Plasmodium*-derived components [known as pathogen-associated molecular patterns (PAMPs)], including hemozoin, glycosylphosphatidylinositol (GPI) anchors, and immunostimulatory nucleic acid motifs, and host-derived damage-associated molecular patterns (DAMPs), including uric acid, microvesicles, and haem, through pathogen-recognition receptors (PRRs) (Gazzinelli and Denkers, 2006; Figueiredo et al., 2007; Schroder and Tschopp, 2010; Takeuchi and Akira, 2010; Barbalat et al., 2011; O’neill et al., 2013; Gallego-Delgado et al., 2014; Mantel and Marti, 2014). During liver stage, *Plasmodium* inside hepatocytes are detected by macrophages and dendritic cells (DCs) through the interaction of parasite RNA with melanoma differentiation-associated protein 5 (MDA5), resulting in production of MDA5-MAVS-IRF3/IRF7-mediated IFN-I (Combes et al., 2004; Couper et al., 2010). At the blood stage, the GPI anchors from *P. falciparum* could stimulate pro-inflammatory responses by macrophages through recognition via TLR1/TLR2 or TLR2/TLR6 and to a much lesser extent through TLR4 (Krishnegowda et al., 2005; Walther et al., 2006; Clark et al., 2008; Riley and Stewart, 2013; Singh and Daneshvar, 2013), resulting in several downstream signaling pathways activation, including MAPK (JNK, p38, and ERK) and NF-κB signaling (Zhu et al., 2005). Hemozoin-combined genomic DNA (gDNA) induces TLR9 translocation on mouse DCs and macrophages and human B lymphocytes, subsequently initiates the activation of NF-κB and MAPK signaling pathways, as well as the release of chemokines and cytokines (Coban et al., 2005; Coban et al., 2010). On the other hand, accumulated studies demonstrate that hemozoin-gDNA complex could also induce NLRP3/AIM2 inflammasomes (Shio et al., 2009; Kalantari et al., 2014). Besides, our results show that the activation of NLRP3/AIM2 dependent inflammasome in plasmacytoid DCs (pDCs), conventional DCs (cDCs), and macrophages during *P. yoelii* YM infection (Yu et al., 2018). We also demonstrate that the *P. yoelii* YM gDNA initiates low level of IFN-I induction via cGAS-STING-TBK1-IRF3 (Yu et al., 2016). Our and other studies indicate that during both the liver and blood stage, parasites RNA activates MDA5-MAVS-mediated IFN-I production with murine parasites (such as *P. yoelii* and *P. berghei*) or human *Plasmoidums* (such as *P. falciparum*) treatment in DCs and macrophages (Baccarella et al., 2013; Liehl et al., 2014; Wu et al., 2014; Yu et al., 2016). In addition, our and other studies denote that the RNA of blood stage parasites induces IFN-I responses through TLR7 signaling pathway (Torgler et al., 2008; Baccarella et al., 2013; Soulat and Bogdan, 2017; Ding et al., 2018). Uric acid, released in large amounts by dying cells, is a byproduct of purine metabolism (Kordes et al., 2011). The pathogenic role of uric acid arises from NLRP3 activation and the inflammatory responses during malaria (Artavanis-Tsakonas and Riley, 2002; Baratin et al., 2007; Griffith et al., 2009; Chen et al., 2014). Host cell-derived microvesicles and haem also trigger inflammatory responses and pathogenesis of malaria (Couper et al., 2010; Ferreira et al., 2011; Mantel and Marti, 2014).

### Innate Immunity

The innate immune responses provide a powerful front line defense against invasive malaria by inhibiting parasite growth and initiating the development of adaptive immunity (Deroost et al., 2016; Thaiss et al., 2016). During the liver stage of malaria infection, recent studies have demonstrated that sporozoites can be phagocytized by neutrophils, which are usually the first circulating cells to respond to an invading *Plasmodium* sporozoites through a mosquito bite (Segal, 2005; Peters et al., 2008; Hopp and Sinnis, 2015). At the blood stage infection, neutrophils clear merozoites via phagocytosis by producing reactive oxygen species (ROS) and other antimicrobial products or by formatting neutrophil extracellular traps (NETs) (Kumaratilake and Ferrante, 2000; Baker et al., 2008; Dale et al., 2008; Dupre-Crochet et al., 2013; Kapelski et al., 2014; Manfredi et al., 2018). The monocytes/macrophages are also important in eliminating parasite to protect host from disease (Chua et al., 2013). Kupffer cells play a pivotal role in preventing the severity of malaria and the release of the parasite into the bloodstream (Tweedell et al., 2015; Tweedell et al., 2018). During the blood stage of malaria, the circulating monocytes are pivotal to control parasitemia by phagocytose merozoite and asexual infected RBCs (iRBCs), as well as by increased inflammatory cytokines (IFN-α, IFN-γ, TNF-α, and IL-6) and chemokines (CCL2, CCL3, CCL4, and CXCL10)  (Huang et al., 2014; Colborn et al., 2015; Bansal et al., 2016; Hommel et al., 2018). Besides, the splenic macrophages play a key role in reducing blood stage parasitemia by phagocytosing iRBCs and producing reactive oxygen intermediates (Sponaas et al., 2009).

Type I IFNs play important roles in controlling malaria infection. An early spike of IFN-I is protective against some *P. yoelii* or *P. berghei* models (Liehl et al., 2014; Wu et al., 2014; Yu et al., 2016). Although the mechanisms remain largely unknown, studies have also shown that chronically high levels of IFN-I inhibit T cell activation, IFN-γ production and humoral immunity; as well as promote DCs death (Haque et al., 2011; Haque et al., 2014; Tamura et al., 2015; Montes De Oca et al., 2016; Zander et al., 2016). During liver stage of malaria infection, IFN-I could be induced by sporozoites in hepatocytes through cytosolic sensing of RNA (Liehl et al., 2014; Miller et al., 2014). At the blood stage, type I IFNs are the earliest cytokines produced by pDCs via TLR7-MyD88-IRF7 signaling pathway. By using lethal model of *P. yoelii* YM infection, our studies have showed that production of early type I IFNs are mediated by TLR7-MyD88-IRF7 signaling pathway and cytosolic sensing mechanisms, which include AIM2/NLRP3-Caspase1-IL-1β-TRAF3-TBK1-IRF3, cGAS-STING, and MDA5-MAVS associated TBK1-IRF3 signaling pathways (Yu et al., 2016; Yu et al., 2018). We further identified SOCS1, expressed in response to cytosolic sensing mechanisms, as a vital regulator to inhibit TLR7-MyD88-dependent IFN-I signaling (Yu et al., 2018). Type I IFNs contribute to the killing of parasite-infected hepatocytes by priming efficient cytokines and chemokines as well as activating γδT, T, natural killer (NK), and NKT cells to induce IFN-γ and other inflammatory cytokines production (Mcnab et al., 2015). The IFN-γ is an important effector that contributes to activating immune cells and indirectly eliminating parasite-infected cells (Liehl et al., 2014; Miller et al., 2014; Stegmann et al., 2015).

### Bridging Innate and Adaptive Immunity

DCs exist in all clinically relevant sites related to the life stage of the malaria parasites and play a vital role in bridging the innate and adaptive immune system (Wykes and Good, 2008). Upon taking up foreign antigens or infecting by malaria parasites, DCs undergo a process of maturation and efficiently present antigen to pathogen-specific T cells via major histocompatibility complex (MHC) surface molecules (Stevenson et al., 1995; Stevenson and Riley, 2004; Orsini et al., 2012). Besides, the DCs secrete several cytokines and chemokines to recruit other immune cells and regulate T and B cells responses, ultimately resulting in clearance of malaria parasites (Wykes et al., 2007; Gowda et al., 2012; Orsini et al., 2012; Wu et al., 2014). An important mechanism for mice to resist *Plasmodium* infection is that the production of IL-12 in DCs, which then initiates the NK cells release IFN-γ to polarize CD4^+^ T helper cell 1 (Th1) (Stevenson et al., 1995; Stevenson and Riley, 2004; Wykes et al., 2007; Gowda et al., 2012). The CD4^+^ Th1 cells evoke effector responses and maintain the memory T cell pool to protect host from *Plasmodium* infection for a long-term (Da Silva et al., 2013). The DCs can be roughly divided into pDCs and cDCs population according to the expression of CD11c and CD123 (Macri et al., 2018). pDCs are the main sources of IFN-α, while cDCs are specifically used to prime and present antigens to T cells (Orsini et al., 2012; Macri et al., 2018). Besides, our studies suggest that pDCs, cDCs, and macrophages are required for generating IFN-I responses against *P. yoelii* YM infection in a stage-specific manner (Yu et al., 2016).

### Adaptive Immunity

Upon DCs presenting processed antigens to naïve T cells, adaptive immunity is activated. Parasite-specific cytotoxic CD8^+^ T cells are essential for the liver-stage protection upon recognition of *Plasmodium* antigens present on MHC class I expressed by DCs and infected hepatocytes (Sedegah et al., 1992; Doolan et al., 1997; Doolan et al., 2003). Yet, at the blood stage of malaria infection, iRBCs have lost the ability to express MHC-I leading to little contribution of CD8^+^ T cell-mediated cytotoxicity to control malaria infection (Kumar and Miller, 1990; Vinetz et al., 1990; Miyakoda et al., 2012). Besides, the function of CD8^+^ T cells in controlling cerebral *Plasmodium* in human is still controversial (Hunt et al., 2010; White et al., 2010). CD4^+^ T cells can be activated by specific polarized cytokines to differentiate into functionally diverse subsets. CD4^+^ Th cells are able to target MHC class II molecules, and play a crucial role in orchestrating innate and adaptive immunity during malaria infection (Roestenberg et al., 2009; Seder et al., 2013; Ishizuka et al., 2016; Mordmuller et al., 2017). IFN-γ and IL-2 are the CD4^+^ Th1-associated cytokines (Shear et al., 1989; Su and Stevenson, 2000; Horowitz et al., 2010). During blood stage *Plasmodium* infection, IFN-γ is essential to activate macrophages and tune class-switch recombination in parasite-specific B cells to evoke antibody response, while IL-2 is critical for activating NK cells (Su and Stevenson, 2000; Jaramillo et al., 2003; Horowitz et al., 2010). The contribution of CD4^+^ Th2 cells remains unknown in anti-malaria immunity (Perez-Mazliah and Langhorne, 2014; Coomes et al., 2015; Walker and Mckenzie, 2018). The T follicular helper (Tfh) cells broadly express the chemokine receptor CXCR5, the transcriptional repressor BCL-6, and the inhibitory receptor programmed cell death protein 1 (PD-1) (Vinuesa and Cyster, 2011). Multiple reports have suggested that Tfh cells, locating at germinal center (GC), can promote protective antibody responses against malaria via providing selection, survival and maturation signal to differentiate GC B cells (Obeng-Adjei et al., 2015; Ryg-Cornejo et al., 2016; Figueiredo et al., 2017; Perez-Mazliah et al., 2017). However, in human malaria, unlike GC Tfh cells, the subsets of circulating Tfh (cTfh) cells play diverse role in anti-malaria immunity. Th1-cTfh cells exhibit a negative role in eliminating parasite, while Th2-cTfh positively correlates with functional antibodies in anti-malaria immune responses (Crotty, 2019; Chan et al., 2020). The Th17 subset is expanded and meaningfully protects host from malaria infection through supporting GC reactions as well as CD8^+^ T cell responses (Wei et al., 2007; Moretto et al., 2017). Another subsets CD4^+^-derived T cells, including those expressing IL-27 and IL-10, appear to inhibit parasite control and protective immunity during *Plasmodium* infection (Couper et al., 2008; Freitas Do Rosario et al., 2012; Gwyer Findlay et al., 2014; Kimura et al., 2016). Treg cells are a subset of CD4^+^ T cells that specifically express the transcription factor FOXP3. In the clinical studies, the Treg cell populations expand after malaria infection. In addition, Treg cell frequency was positively correlated with parasite load (Jangpatarapongsa et al., 2008; Torcia et al., 2008; Hansen and Schofield, 2010). Several experimental studies have shown that during blood-stage malaria, Treg cells block effective interactions between Tfh and B cells in GC responses during blood-stage infection (Abel et al., 2012; Kurup et al., 2017).

During a primary infection, antibody-independent immune mechanism can usually limit the severity of malaria infection. However, the B cells and antibodies are essential for complete parasite clearance and providing protection against reinfection, which are coordinated by CD4^+^ Th1 cells via indirectly targeting iRBCs, lacking expression of MHC molecules (Mendis and Targett, 1979; Osier et al., 2008; Fowkes et al., 2010; Perez-Mazliah et al., 2015; Stone et al., 2018). Hence, studying B-cell responses to *Plasmodium* at the monoclonal level has great potential for the development of effective vaccines and therapies (Imkeller et al., 2018; Murugan et al., 2018; Alanine et al., 2019; Mcleod et al., 2019). The antibody-dependent immune responses target circulating parasites and infected host cells expressing parasite antigens on their surfaces (Doolan et al., 2009). Several studies have indicated that the protective antibody titers could not be efficiently induced to against malaria both at liver- and blood-stage, which arise from no time for maturation of long-lived antibody-secreting plasmablasts and highly variable antigen-mediated immune escape of merozoites, respectively (Hoffman et al., 1987; Wahlgren et al., 2017; Aliprandini et al., 2018). Besides, multiple studies hypothesized that B cell responses might be suboptimal or dysfunctional after malaria parasites infection, resulting in defective long-lasting humoral memory (Marsh et al., 1989; Gupta et al., 1999; Silveira et al., 2018). To support above assumption, a series of field studies have indicated that *Plasmodium*-specific antibody responses retain a much shorter lifespan than their homologous memory B cell responses, especially in children (Crompton et al., 2010; Weiss et al., 2010; Ndungu et al., 2012; Ndungu et al., 2013). Recent advance has suggested that during the blood stage, the short-lived plasmablasts expand to constrain GC-dependent humoral immunity both in human and mice (Vijay et al., 2020). Interestingly, several reports have shown that antibodies could activate the complement system to against parasites (Ratelade and Verkman, 2014; Kurtovic et al., 2020). Antibody-mediated complement activation can protect and enhance antibody efficacy by exploiting Fc-mediated neutralization and lysis of target cells. Notably, complement-mediated lysis is strongly observed in human, guinea pig, and rat serum, except for mouse. Furthermore, The AMB, T-bet-expressing B cells, expands in blood stage during *Plasmodium* infection (Weiss et al., 2009). The function of T-bet^+^ AMB (atypical memory B cells) on host protection from malaria infection is still undetermined (Barnett et al., 2016; Rivera-Correa et al., 2017).

## Regulators In Anti-Malaria Immune Responses

**Figure 2:**
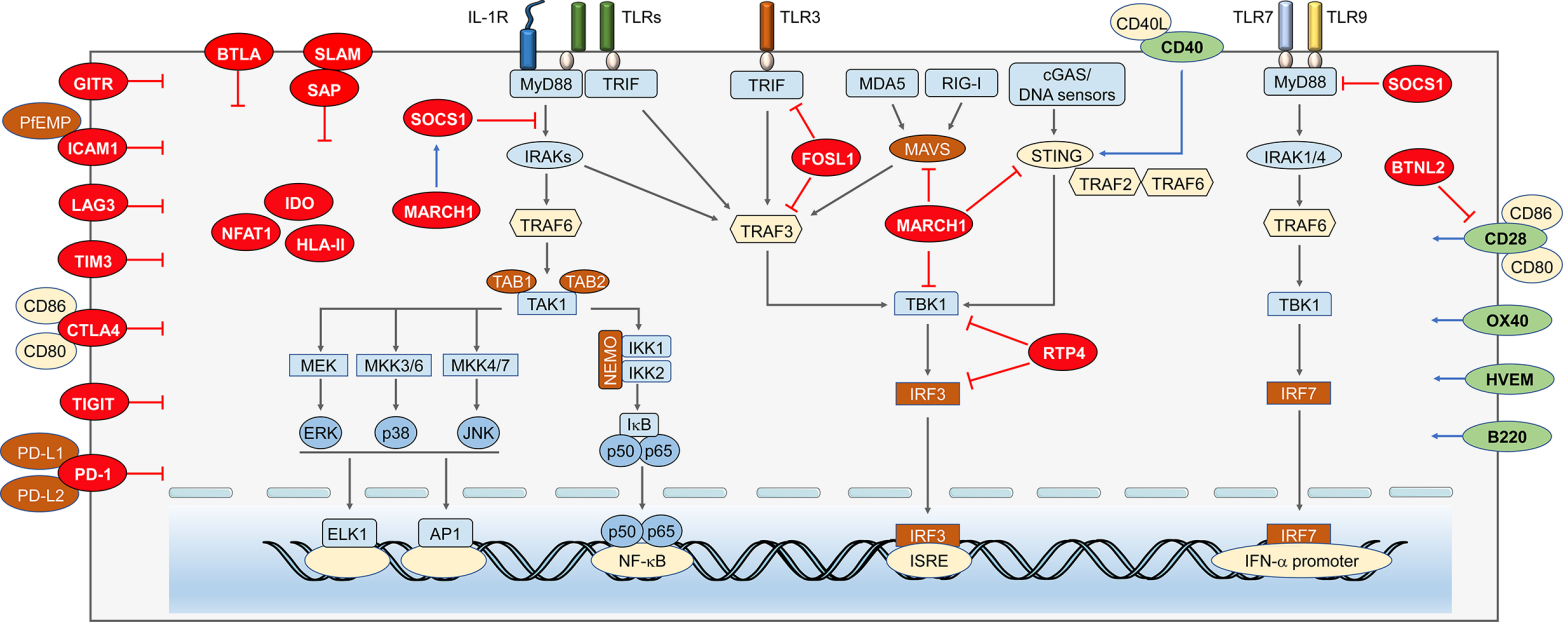
Immune regulators in anti-malaria immunity. Anti-malaria immunity is tightly regulated by cytosolic and cell surface molecules at multiple levels. *Negative regulators within cytosol.* As a negative molecule binds to MyD88, SOCS-1 can not only suppress the NF-κB and MAPKs signaling, but also inhibits MyD88-mediated IFN-I signaling. MARCH1 can reduce IFN-I through increasing expression of SOCS-1. Besides, its target proteins, MAVS, STING, and TBK1, possibly are degraded through ubiquitination. FOSL1 inhibits the K63 ubiquitination of TRAF3 and TRIF and disrupts TBK1/TRAF3/TRIF complexes formation, which results in reduced phosphorylation of IRF3 and suppresses IFN-I production. RTP4 inhibits the expression of TBK1 and IRF3 and/or activation by binding with TBK1 to reduce IFN-I production. *Negative regulators on the cell membrane*. Binding of ICAM1 and PfEMP leads to static adhesion of parasite and evades immune clearance. LAG3 inhibits T cell responses and antibody secreting B cell responses. CTLA-4 expressed on activated T cells could suppress the CD4^+^ Th and humoral immune responses. TIM3 can inhibit T lymphocytes, NK cells, and macrophages responses. TIGIT proteins mainly suppress the function of T cell and NK cell. The activation of PD-1 pathway dramatically inhibits TCR-mediated proliferation and function of T cells. *Positive regulators on the cell membrane.* CD40 could compete with STING to bind TRAF2/3 and/or TRAF6 to reduce STING ubiquitination, which leads to increased expression of IFN-I. CD28 can enhance CD4^+^ T cell responses, but the CD28-B7 signals can also be inhibited by BTNL2. OX40 enhances *Plasmodium* specific CD4 T cell activity. B220 expressed on the surface of B and T cells can help promoting the maturation of both T and B cell.

Anti-malaria immune responses are tightly modulated to maintain host defense and immune balance. These include positive regulators to accelerate the immune responses and negative regulators to attenuate the immunity. Over the past decades, many immune regulators have been identified (**Figure 2**), which could lead to beneficial or detrimental outcomes for the host. Understanding how anti-malaria immune responses are regulated by these regulators will obviously facilitate the development of new effective vaccines and therapies. Next, we summarize the current knowledge of the positive and negative regulators involved in anti-malaria immune responses and discuss the mechanism by which these regulators orchestrate host immunity against malaria.

### Positive Regulators

#### CD40

CD40 (or TNFRSF5), a member of TNF receptor superfamily, is broadly expressed on the surfaces of many cell types, including monocytes, DCs, B cells, endothelial cells, and epithelial cells (Van Kooten and Banchereau, 2000). The signal transduction mediated by CD40-CD40L interaction could activate NF-κB, STAT3, MAPK, and other kinases (Van Kooten and Banchereau, 2000; Elgueta et al., 2009). Accumulated studies have indicated that CD40 is responsible for promoting cellular and humoral adaptive immunity and inflammatory responses (Van Kooten and Banchereau, 2000; Benveniste et al., 2004; Elgueta et al., 2009; Carlring et al., 2011). Besides, interaction of CD40 and CD40L between the DCs and CD4^+^ T cells triggers DCs to activate cytotoxic CD8^+^ cells (Bennett et al., 1998; Ridge et al., 1998; Schoenberger et al., 1998). Recent advances suggested that CD40-CD40L ligation could initiate the activation TRAF2/3 mediated NF-κB pathways and production of IRF1 to eventually induce IFN-β expression (Stirnweiss et al., 2010; Moschonas et al., 2012). CD40 was reported to help eliminating the malaria parasites to reduce the severity of disease (Inoue et al., 2012; Murray et al., 2015; Gramaglia et al., 2017; Parmar et al., 2018). Yao et al. found that CD40 could compete with STING to bind TRAF2/3 and/or TRAF6 to reduce STING ubiquitination, leading to dampen STING degradation and increase STING protein level (Yao et al., 2016). The increase of CD40 expression by *P. yoelii* N67 infection could enhance the protein level of STING, which in turn promotes the IFN-I production during early stage of infection and results in better host survival (Yao et al., 2016). It is also indicated that iRBCs, parasite DNA/RNA, and various TLR ligands, could induce CD40 expression. After malaria infection, a signaling axis of TLR recognition and signaling is established, leading to increase CD40 and STING levels, enhance IFN-I production, and prolong host survival (Yao et al., 2016).

#### CD28

CD28 constitutively expresses on T cells surface (Gross et al., 1992). The CD80/86-CD28 interaction between APCs and T cells is an important costimulatory signal for T cell activation, which serves as a crucial second signal for T cell activation along with the MHC-TCR signaling (Carreno and Collins, 2002). CD28 could promote transcription signaling transduction, activation of cytokines or cytokine receptors, as well as activation and differentiation of naïve CD4^+^ and CD8^+^ T cells, to protect host from a variety of pathogens (Sharpe and Freeman, 2002; Elias et al., 2005; Butty et al., 2007; Cong et al., 2014; Niknam et al., 2017). During malaria infection, CD28 plays an essential role in enhancing CD4^+^ T cell responses and CD4^+^ T cell-driven antibody class switch (Falanga et al., 1984; Langhorne et al., 1989; Sanchez-Torres et al., 2001). Besides, CD28-CD80/CD86 signals increase the survival and proliferation of γδ T cells to reduce severity of malaria infection (Dieli et al., 2001; D’ombrain et al., 2007; Ribot et al., 2012).

#### OX40

OX40 receptor, transiently expressed on T cells, is an important co-stimulatory molecule that interacts with TCRs and MHC complexes on APCs (Croft, 2010). Previous reports have denoted that OX40 promotes proliferation and survival of T cell, as well as differentiation of CD4^+^ T cells into Th1, Th2 and Tfh cell subsets; and reportedly reverses the hyperactivity of CD4^+^ T cell (Walker et al., 1999; Bansal-Pakala et al., 2001; Croft, 2010). Recent studies have demonstrated that OX40 would enhance *Plasmodium* specific CD4^+^ and CD8^+^ T cell activity, as well as parasite specific humoral immunity (Zander et al., 2015; Zander et al., 2017; Othman et al., 2018; Xia et al., 2018).

#### Other Positive Regulators

Herpes virus entry mediator (HVEM), a co-stimulatory receptor, expresses on the surface of B cells, T cells, endothelial cells and mast cells (Chang et al., 2005; Guo et al., 2015; Sibilano et al., 2016). Upon bacterial and viral infections, HVEM-mediated signaling pathway enhances T cell expansion and is necessary for persistence of memory T cell (Soroosh et al., 2011; Flynn et al., 2013; Steinberg et al., 2013; Desai et al., 2017). Recent findings of Muscate et al. indicated that HVEM is required to provide pro-survival signals for stabilizing CD8^+^ T effector cells during malaria infection (Muscate et al., 2018). During *P. berghei* ANKA infection, the HVEM-CD160 ligation is crucial for the delicate regulation of stimulatory and inhibitory signals in CD8^+^ T cells.

B220 is a high-molecular mass alternative splicing isoform of the tyrosine phosphatase CD45 (Renno et al., 1998). Its expression is found not only in B cell, including precursors, mature and memory B cells, but also in a subset activated T cells and pDCs (Marvel and Mayer, 1988; Hathcock et al., 1992; Renno et al., 1998; Rodig et al., 2005). Several studies have suggested that malaria exposure increased frequencies of B220, which is essential for promoting both T and B cells maturation (Asensi et al., 1990; Bakir et al., 2006; Niikura et al., 2008; Kanda et al., 2010; Bao et al., 2015; Ubillos et al., 2017).

### Negative Regulators

#### PD-1

Unlike other CD28 family members, the expression of PD-1(CD279) could be induced on activated monocytes, NK cells, DCs, myeloid cells, CD4^+^ T cells, CD8^+^ T cells, B cells, and a subset of thymocytes through TCR or BCR mediated signaling; as well as being augmented by stimulation of tumor necrosis factor (TNF) (Agata et al., 1996; Nakae et al., 2006; Okazaki and Honjo, 2006). The activation of PD-L1/PD-1 pathway dramatically inhibits TCR-mediated proliferation and function of CD4^+^ and CD8^+^ T cells (Freeman et al., 2000; Latchman et al., 2001; Rodig et al., 2003; Grosso et al., 2009). Several studies have demonstrated that pathogens including bacteria, viruses (such as HIV), protozoan parasites (including *Plasmodium*), and tumor cells could exploit above PD-1 inhibitory function on T cells to evade host adaptive immunity (Day et al., 2006; Horne-Debets et al., 2013; Gubin et al., 2014). In malaria infection, the number and function of parasite-specific CD4^+^ T cells (including Th1 and Tfh), CD8^+^ T cells, and memory T cells are significantly inhibited by PD-1 with decelerated parasite clearance (Horne-Debets et al., 2013; Karunarathne et al., 2016; Liu T. et al., 2018; Pan et al., 2020). However, the inhibition of B cell function by PD-1 is controversial. During *P. chabaudi* infection, PD-1 elimination does not improve primary B cell responses (Horne-Debets et al., 2013). In contrast, multiple studies have indicated that PD-1 deficiency substantially promoted expansion of GC B cells and humoral immunity in ITV (infection treatment vaccine)–immunized mice (Liu T. et al., 2015; Liu T. et al., 2018); and PD-1 elimination significantly increased long-lived plasma cells by *P. berghei* ANKA infection (Pan et al., 2020).

#### SOCS1

SOCS1 is one of the members of suppressors of cytokine signaling (SOCS) family, which regulates signal transduction pathways triggered by activation of cytokine and hormone receptors (Machado et al., 2006; Dimitriou et al., 2008). *Socs1^−/−^* mice die within 3 weeks after birth, due to the high level of JAK/STAT-mediated inflammation, which could be significantly inhibited by SOCS1 (Naka et al., 1997; Starr et al., 1997; Naka et al., 1998; Starr et al., 1998), and this mice are highly susceptible to sepsis comparing to WT mice (Kinjyo et al., 2002; Nakagawa et al., 2002). *Socs1^−/−^* macrophages produce high amount of proinflammatory cytokines and nitric oxide after LPS and CpG stimulation. Moreover, SOCS1 seems to inhibit LPS- but not TNF-mediated NF-κB signaling through promoting the degradation of IRAK and NF-κB (Dimitriou et al., 2008).

Our studies have demonstrated that SOCS1 could interact with MyD88 to inhibit TLR7-MyD88-IRF7 dependent IFN-I signaling after lethal malaria infection (Yu et al., 2016; Yu et al., 2018; Cai and Yu, 2020). During malaria infection, *Plasmodium* gDNA and RNA activate cGAS/STING and MDA5/MAVS to induce IRF3-dependent SOCS1 production, respectively, which, in turn, bind to MyD88 to suppress MyD88-IRF7 dependent IFN-I response, leading to fast parasite growth and host death (Yu et al., 2016). Furthermore, *Plasmodium* gDNA, RNA, and hemozoin also activate inflammasome signaling and lead to production of IL-1β, which subsequently induces SOCS1 expression to inhibit MyD88 dependent IFN-I production (Yu et al., 2018). Moreover, a recent study further illustrated the delicate cross-regulation between several IFN-I signaling pathways and inflammasome signaling pathways during lethal *P. yoelii* YM infection. Inflammasome-, MAVS-, and STING-mediated signaling pathways have diverse impacts on regulating MyD88-dependent IFN-I responses after *P. yoelii* YM infection; and the dosage of *P. yoelii* YM has significant effect on the differences of resistance among inflammasome, MAVS, and STING deficiency (Cai and Yu, 2020).

It has been reported that SOCS1 could simultaneously inhibit MyD88-dependent IFN-I production and downstream IFN-dependent production of IFN-γ. However, SOCS1 deficiency could not protect host from infection in *Myd88^−/−^* mice, suggesting that SOCS1 controls the host resistance to malaria mainly through MyD88-mediated IFN-I production (Yu et al., 2018). Importantly, SOCS1 could be induced by IRF3-dependent signaling, LPS, and CpG DNA, as well as the downstream IFN-stimulating pathway, indicating a negative-feedback regulatory mechanism to sustain its inhibitory function.

#### FOSL1

FOSL1 belongs to a gene family that encode proteins containing leucine zippers (Macchia et al., 2012), which are identified as regulators in trophoblast migration and invasion (Renaud et al., 2014). The FOSL1 contributes to several cellular processes, including proliferation, differentiation, and apoptosis via serving as a member of transcription factors and musculoaponeurotic fibrosarcoma (MAF) (Macchia et al., 2012). It has been reported that FOSL1 is a key downstream effector of the phosphatidylinositol 3-kinase (PI3K)/AKT signaling pathway to control *Mmp9* gene expression and trophoblast lineages development (Kent et al., 2011). In addition, FOSL1 also plays a pivotal role in diverse cancers (Young and Colburn, 2006).

FOSL1 is normally expressed in the nucleus, while Cai et al. showed that cGAS-STING-, TRIF-, or RIG-I/MDA5-MAVS-mediated IFN-I production could evoke the “translocation” of FOSL1 into the cytoplasm after iRBCs stimulations. The cytoplasmic FOSL1 inhibits the formation of TBK1/TRAF3/TRIF complexes by suppressing K63 ubiquitination of TRAF3 and TRIF. The inhibition of FOSL1 on TBK1 complexes leads to suppression of IFN-I production (Cai et al., 2017). Hence, these reports identified FOSL1 as a negative regulator of IFN-I production during early infection and liver stages, resulting in fast parasite growth and host death (Liehl et al., 2014; Wu et al., 2014; Yu et al., 2016; Yu et al., 2018).

#### MARCH1

Membrane-associated ring-CH–type finger 1 (MARCH1) is a member of membrane-bound E3 ubiquitin ligases expressed mostly by DCs and B cells (Ohmura-Hoshino et al., 2006; Wu et al., 2020). The MARCH1 production is initiated by IL-10 after TLR4 and CD40 signaling (Galbas et al., 2012; Mittal et al., 2015). Besides, stimulating the monocyte-derived macrophages could enhance endogenous expression of MARCH1 (Zhang et al., 2019b). Previous studies, focusing on MARCH1 function, have shown that MARCH1 ubiquitinates CD86 and MHC-II molecules for degradation to down-regulate adaptive immunity; and may influence CD28, CTLA4, and PD-L1 signaling by regulating relative protein levels of CD80/CD86 on antigen-presenting cells (APCs) (Ishido et al., 2009; Samji et al., 2014; Ablack et al., 2015).

Wu et al. have recently indicated that upon *P. yoelii* N67 or *P. yoelii* YM treatment, mice deficient in *March1* had significant better survival rates than WT mice, indicating the negative function of MARCH1 in generating protective immunity against malaria infection (Liehl et al., 2014; Wu et al., 2014; Yu et al., 2016). Clustered with several ISGs in the Ts-eQTL analysis, MARCH1 deficient mice produce higher amount of IFN-I than WT mice in response to cGAMP and poly(I:C), accompany with increased protein levels of STING, MAVS, TRAF3 and TRAF6 in uninfected condition. However, reduced production level of IFN-I was observed in MARCH1 deficient mice during early malaria infection. The mechanism by which MARCH1 regulates IFN-I remains obscure and several factors may include increased expression of genes encoding SOCS1, SOCS3, SIKE1, CACTIN, TRIM24, IL-10RA, USP18, and mir-21 that are known to suppress IFN-I responses (Huang et al., 2005; Yu and Hayward, 2010; Liu D. et al., 2015; Arimoto et al., 2017) and changes in DCs, Macs, and other cell populations can may also affect the levels of proteins critical for IFN-I production (Wu et al., 2020). In addition, MARCH1 deficiency significantly increased numbers of both CD86^+^ DCs population, which could promote CD86-CD28 interaction and T cell activation, leading to enhanced Th1-mediated response and IFN-γ production (Wu et al., 2020).

#### RTP4

Receptor transporter family is known to promote cell surface expression of a group of G-protein-coupled receptors (GPCR). RTP4, a member of the RTP family, can be induced after viral infection (Nair S. R. et al., 2017; Dang et al., 2018). In addition, RTP4 plays an important role in mediating pain relief, bitterness, or odor sensing (Behrens et al., 2006; Decaillot et al., 2008). Su’s group has suggested that the IFN-I and IFN-I pathways can induce RTP4 expression after parasite infection. Besides, RTP4 significantly inhibits IFN-I response through inhibition of TBK1 and IRF3 expression and activation by binding with TBK1 to reduce IFN-I production. Inhibition of RTP4 expression may help reduce parasitemia and help to alleviate symptoms of cerebral malaria (CM) and other diseases with neuropathology (He et al., 2020). Besides, Su’s group also demonstrated that *P. yoelii* HECT-like E3 ubiquitin ligase (*Py*HEUL) encoding by malaria genome may indirectly affect host immune response and parasite infection by regulating the expression levels of proteins, such as merozoite surface protein 1 (MSP1) and/or cytoadherence-linked asexual gene 2 (CLAG2) (Nair S. C. et al., 2017).

#### Other Negative Regulators

Butyrophilin-like 2 (BTNL2) is a butyrophilin family member. Previous studies have demonstrated that BTNL2 inhibits T cells proliferation and is involved in a variety of autoimmune diseases (Lin et al., 2015; Tian et al., 2019). Besides, BTNL2 promotes Tregs generation through modifying B7/CD28 signaling (Swanson et al., 2013). Recent study has shown that BTNL2 also dampens T cells proliferation and activation in the *P. berghei* model of experimental cerebral malaria (ECM) (Subramaniam et al., 2015).

Indoleamine 2,3-dioxygenase (IDO) is induced by pro-inflammatory mediators, such as endotoxin and IFN-γ, in several tissues. IDO is a tryptophan-degrading enzyme; and has been identified an inhibitory function on proliferation of facultative intracellular pathogens and tumor cells (Taylor and Feng, 1991). In addition, IDO could suppress responses of T cells and promote tolerance (Mellor and Munn, 2004). Several studies have denoted that IDO is involved in CM (Medana et al., 2002; Medana et al., 2003; Mitchell et al., 2005).

Nuclear factor of activated T cell 1 (NFAT1) is a member of NFAT transcription factors, which are required for regulating the activation and differentiation of T cells (Macian, 2005). Multiple field reports, including malaria infection, have indicated that NFAT1-regulated gene expression is essential in efficient Treg-mediated CD4^+^ T cell suppression (Soto-Nieves et al., 2009; Shin et al., 2014; Ames et al., 2017; Kadziolka et al., 2017).

CD160 is expressed by NK, NKT, CD8^+^ T, γδ T, iIELs, ILC1, mast, and a minority of CD4 ^+^ T cells (Maeda et al., 2005; Ortonne et al., 2011; Fuchs et al., 2013). The function of CD160 on T cells remains controversial, some studies have suggested that CD160 is a co-inhibitory molecule, while others have indicated that it has co-stimulative functions that promote proliferation and cytotoxicity of T cells, as well as inflammatory cytokine production (Le Bouteiller et al., 2002; Barakonyi et al., 2004; Cai et al., 2008). Strikingly, during malaria, CD160 is crucially involved in restricting CD8^+^ T cytotoxicity and IFN-γ production (Muscate et al., 2018).

B and T lymphocyte attenuator (BTLA) (CD272), a co-inhibitory receptor, is expressed by most leukocytes (Muscate et al., 2018). The BTLA is required for maintenance of peripheral tolerance by inhibiting lymphocytes activation. In malaria model, BTLA dampens innate immune responses and T/B cell-mediated immune response to malaria infection (Sun et al., 2009; Vendel et al., 2009; Adler et al., 2011).

Once T cells are activated by CD28-CD80/CD86 signal, they enhance expression of cytotoxic T lymphocyte antigen-4 (CTLA-4, also named CD152), which is another receptor for CD80/CD86. CTLA-4 is shown to be involved in maintenance of peripheral tolerance; inhibition of immune response against tumors and infectious diseases; as well as increased severity of autoimmune diseases (Gregor et al., 2004; Martins et al., 2004).

T-cell immunoglobulin- and mucin-domain-containing molecule 3 (TIM-3) is expressed by monocytes, macrophages, NK cells, DCs, and Th1 and Tc1 lymphocytes (Sakuishi et al., 2011). The binding of TIM-3 and galectin-9 negatively regulates T-cell and NK-cell activation in many diseases (Sabatos et al., 2003; Ju et al., 2010; Bi et al., 2011; Hou et al., 2012). Accumulated findings indicated that TIM-3 was responsible for inhibiting T lymphocytes (CD4^+^ Th1/Th2, CD8^+^, and γδ T cells), NK cells, and macrophages responses to malaria treatment (Hou et al., 2016; Hou et al., 2017; Sabins et al., 2017; Schofield et al., 2017; Otterdal et al., 2018).

T cell immunoglobulin and ITIM domain (TIGIT) is expressed on the surface of NK cells and T cells (Yu et al., 2009). It has reported that TIGIT as an inhibitor controls the function of NK cells, CD4^+^ T cells, and CD8^+^ T cells (Johnston et al., 2014). During *P. berghei* ANKA infection, the upregulation of TIGIT proteins results in inhibition on T and NK cells (Villegas-Mendez et al., 2016; Zhang et al., 2019a).

The Signaling Lymphocytic Activation Molecule (SLAM)–Associated Protein (SAP), a small intracellular adaptor protein, could interact with the SLAM family and mediate downstream signaling of these receptors (Cannons et al., 2011). The deficient of SAP promotes the activation of Tfh and GC B cells, as well as IgG response against malaria infection (Perez-Mazliah et al., 2017).

Lymphocyte Activation Gene-3 (LAG-3) is expressed by many cells, including NK cells, T cells, B cells, and tumor infiltrating lymphocytes (Huang et al., 2004). As the T cell receptors, LAG-3 directly and indirectly induces transcriptional changes, which negatively modulates proliferation and pro-inflammatory cytokines expression by virus-specific CD8^+^ T cells (Barber et al., 2006; Blackburn et al., 2009). Butler et al. recently have shown that LAG-3 substantially inhibits T-cell responses and antibody secreting B cell responses to malaria infection (Butler et al., 2011).

Intracellular adhesion molecule 1(ICAM1, also named CD54) belongs to immunoglobulin superfamily; and is expressed by endothelial cells and leukocytes (Rowe et al., 2009). Binding of ICAM1 and malaria ligands, members of the PfEMP1 family, leads to static adhesion of parasite (Chattopadhyay et al., 2004; Springer et al., 2004). This adhesion occludes blood flow, leads to inflammation, and evades immune clearance (Lennartz et al., 2017; Lennartz et al., 2019).

The MHC [human leukocyte antigen (HLA) in humans] class II heterodimers are major participants in generating an immune response against microorganisms for providing exogenous peptides to activate and differentiate CD4^+^ T cells (Rothbard and Gefter, 1991). Previously, CD4^+^ Th1, Th2, and Th17 cells have been reported to be essential for coordinating the cellular and/or humoral response to clear pathogens, while the Treg cells downregulate those responses via secretion of suppressive cytokines or through direct cell-cell interactions (Surls et al., 2010; Ma et al., 2012; Issa et al., 2013). Importantly, during malaria infection, the HLA-II molecules evoke expansion and activation of Tregs to suppress antibody production by direct cell-cell interaction with B cells (Storti-Melo et al., 2012; Wijayalath et al., 2014).

Glucocorticoid-induced tumor necrosis factor receptor (GITR) constitutively expresses on cell surface of natural Tregs (Yagi et al., 2004; Belkaid and Rouse, 2005; Nocentini et al., 2007). The interaction of GITR and agonist antibody or GITR ligand (GITRL) appears to abrogate suppressive activity of Tregs (Mchugh et al., 2002; Shimizu et al., 2002; Shevach and Stephens, 2006). However, engagement of GITR promotes proliferation and suppressive function of Tregs (Shevach and Stephens, 2006; Nocentini et al., 2007; Nishioka et al., 2008). In malaria infection, GITR is involved in the escape of parasites from host T cell immune responses (Hisaeda et al., 2005).

## Concluding Remarks

Nearly 160 years post the discovery of *Plasmodium* in 1861, malaria is still a serious threat to global health that accounts for about 0.5 million deaths every year. Although significant progress has been done in most endemic area, eliminating malaria is still halted by emergence of drug resistance. Furthermore, the lack of an effective vaccine has been one of the major limiting factors in the prevention of malaria infection, which mainly due to the incomplete understanding of the underlying mechanism of host-parasite interactions.

During the past 10 years, stepwise progresses have been achieved in understanding the immune responses to malaria infection and their contribution to eliminating parasites. Yet, malaria infection triggers a systemic immune response, which in turn induces an increase in the production of inflammatory cytokines that lead to parasite elimination and/or immune pathology. *Plasmodium* infection induces host balanced responses in which activating signaling for anti-malaria states and promoting immunity counterbalanced by suppressive signaling that limit toxicity and enable chronic infection. Hence, a fine-tuned regulation of immune responses is crucial for developing protective immunity to effectively eliminate malaria parasites and preventing damage to host.

*Plasmodium* also exploits regulatory mechanisms to escape immune responses, including enhancing negative regulators and inhibiting positive regulators. We noticed that much more negative regulators are discovered than the positive regulators due to date, which may indicate that *Plasmodium* infection initiates multiple tools or signaling to evade host immune responses. Advances in these areas would aid the development of malaria vaccines and therapeutics that could selectively target pathogenic regulators while leaving defensive regulators intact. Here, we highlight our studies indicating the crucial role of early spike of IFN-I in protecting host from *Plasmodium* infection, and focus on discussing the regulatory network of immune responses against to malaria infection. Besides, we also emphasize our results demonstrating the importance of regulators mediated tune modulation of immune responses during malaria treatment. The search for more efficient vaccines and novel treatment strategies is a major objective in reducing the burden of malaria. Hence, any effort to control and eradicate malaria requires a better understanding of the contribution and regulation of immune responses to *Plasmodium* infection. In addition, new technologies, including whole genome-wide analysis in a single immune cell level and application of Mass Cytometry would offer promise for a more comprehensive investigation of immune responses and regulations in responses to malaria, and should facilitate the development of new effective vaccines and therapies.

## Author Contributions

CC wrote the manuscript. ZH drew the figures and wrote the manuscript. XY wrote the manuscript and supervised the entire project. All authors contributed to the article and approved the submitted version.

## Funding

This work was supported by grants from the National Science Foundation of China (No. 81801579), Science and Technology Planning Project of Guangzhou (No. 201904010064), Guangdong Basic and Applied Basic Research Foundation (No. 2019B1515120033), Zhujiang Youth Scholar Funding, and the Start-up Fund for High-level Talents of Southern Medical University to XY. This work was also supported by the National Natural Science Foundation of China (No. 81960292) to CC.

## Conflict of Interest

The authors declare that the research was conducted in the absence of any commercial or financial relationships that could be construed as a potential conflict of interest.

## Abbreviations

AIM2, absent in melanoma 2; AP-1, activating protein-1; APCs, antigen presenting cells; ATF, activating transcription factor; BCR, B cell receptor; BTLA, B and T lymphocyte attenuator; BTNL2, Butyrophilin-like 2; cGAS, cyclic GMP-AMP synthase; CCL, The chemokine (C-C motif) ligand; cDCs, conventional dendritic cells; CLAG2, cytoadherence-linked asexual gene 2; CTLA-4, Cytotoxic T lymphocyte antigen-4; CXCL, Chemokine (C-X-C motif) ligand; DAMPs, danger-associated molecular patterns; DC, dendritic cell; ECM, experimental cerebral malaria; ERK, extracellular signal-regulated kinases; FOXP3, Forkhead Box P3; GC, germinal center; gDNA, genomic-DNA; GPCR, G-protein-coupled receptors; GPI, glycosylphosphatidylinositol; HVEM, Herpes virus entry mediator; IDO, Indoleamine 2,3-dioxygenase; IFN-I, type I interferon; IL, interleukin; iRBCs, infected red blood cells; IRAK, IL-1 receptor associated kinase; IRF, IFN regulatory factor; JAK, Janus kinase; JNK, c-Jun N-terminal kinases; LAG-3, Lymphocyte Activation Gene-3; MAF, musculoaponeurotic fibrosarcoma; MAPK, mitogen-activated protein kinases; MARCH1, Membrane-associated ring-CH–type finger 1; MDA-5, melanoma differentiation-associated protein 5; MAVS, mitochondrial antiviral-signaling protein; MHC, major histocompatibility complex; MSP1, merozoite surface protein 1; MyD88, myeloid differentiation factor 88; NETs, neutrophil extracellular traps; NFAT1, Nuclear factor of activated T cell 1; NF-κB, nuclear factor κB; NK cells, natural killer cells; NKT cells, NK T cells; NLRP3, NLR family pyrin domain containing 3; PAMPs, pathogen-associated molecular patterns; PD-1, programmed cell death protein 1; pDCs, plasmacytoid dendritic cells; PI3K, phosphatidylinositol 3-kinase; PRRs, pathogen recognition receptors; PyHEUL, *Plasmodium yoelii* HECT-like E3 ubiquitin ligase; RBCs, red blood cells; ROS, reactive oxygen species; SOCS, suppressors of cytokine signaling; STAT, Signal transducer and activator of transcription; STING, stimulator of IFN genes; TBK1, TRAF family member-associated NF-κB activator (TANK)–binding kinase 1; TCR, T cell receptor; Tfh cells, T follicular helper cells; Th cells, helper T cells; TIGIT, T cell immunoglobulin and ITIM domain; Tim-3, T-cell immunoglobulin- and mucin-domain-containing molecule 3; TLR, Toll-like receptor; TNF, Tumor necrosis factor; TNFRSF5, TNF receptor superfamily member 5; TRAF, tumor necrosis factor receptor-associated factor; Treg cells, regulatory T cells.
